# Biosynthesis of Long-Chain Polyunsaturated Fatty Acids in Marine Gammarids: Molecular Cloning and Functional Characterisation of Three Fatty Acyl Elongases

**DOI:** 10.3390/md19040226

**Published:** 2021-04-16

**Authors:** Alberto Ribes-Navarro, Juan C. Navarro, Francisco Hontoria, Naoki Kabeya, Inger B. Standal, Jan O. Evjemo, Óscar Monroig

**Affiliations:** 1 Instituto de Acuicultura de Torre de la Sal (IATS-CSIC), 12595 Ribera de Cabanes, Castellón, Spain; alberto.ribes@csic.es (A.R.-N.); jc.navarro@csic.es (J.C.N.); hontoria@iats.csic.es (F.H.); 2 Department of Marine Biosciences, Tokyo University of Marine Science and Technology, Konan 4-5-7, Minato, Tokyo 108-8477, Japan; naoki.kabeya@kaiyodai.ac.jp; 3 Department of Fisheries and New Biomarine Industry, SINTEF Ocean, 7010 Trondheim, Norway; inger.b.standal@sintef.no (I.B.S.); Jan.O.Evjemo@sintef.no (J.O.E.)

**Keywords:** *Echinogammarus marinus*, elovl enzymes, LC-PUFA biosynthesis, functional characterisation, gammarids

## Abstract

Long-chain (C_20–24_) polyunsaturated fatty acids (LC-PUFAs) are essential nutrients that are mostly produced in marine ecosystems. Previous studies suggested that gammarids have some capacity to endogenously produce LC-PUFAs. This study aimed to investigate the repertoire and functions of elongation of very long-chain fatty acid (Elovl) proteins in gammarids. Our results show that gammarids have, at least, three distinct *elovl* genes with putative roles in LC-PUFA biosynthesis. Phylogenetics allowed us to classify two elongases as Elovl4 and Elovl6, as they were bona fide orthologues of vertebrate Elovl4 and Elovl6. Moreover, a third elongase was named as “Elovl1/7-like” since it grouped closely to the Elovl1 and Elovl7 found in vertebrates. Molecular analysis of the deduced protein sequences indicated that the gammarid Elovl4 and Elovl1/7-like were indeed polyunsaturated fatty acid (PUFA) elongases, whereas Elovl6 had molecular features typically found in non-PUFA elongases. This was partly confirmed in the functional assays performed on the marine gammarid *Echinogammarus marinus* Elovl, which showed that both Elovl4 and Elovl1/7-like elongated PUFA substrates ranging from C_18_ to C_22_. *E. marinus* Elovl6 was only able to elongate C_18_ PUFA substrates, suggesting that this enzyme does not play major roles in the LC-PUFA biosynthesis of gammarids.

## 1. Introduction

Long-chain (C_20–24_) polyunsaturated fatty acids (LC-PUFAs), including arachidonic acid (ARA, 20:4n-6), eicosapentaenoic acid (EPA, 20:5n-3) and docosahexaenoic acid (DHA, 22:6n-3), are physiologically essential compounds required for normal growth and development of vertebrates [1]. Moreover, the n-3 (or omega-3) LC-PUFAs EPA and DHA have beneficial roles in human health [2]. Since n-3 LC-PUFAs are nearly exclusively produced in marine ecosystems by low trophic marine organisms, such as microbes [3,4,5] and certain invertebrates [6,7], marine products are regarded as unique sources of EPA and DHA in the human diet [8]. Products derived from marine aquaculture have traditionally guaranteed the supply of health-promoting n-3 LC-PUFAs due to the fact that aquafeeds were formulated with high inclusion levels of the so-called “marine ingredients” fishmeal (FM) and fish oil (FO), naturally rich in n-3 LC-PUFAs [9,10]. FM and FO are mostly produced from feed-grade fish species and, with the rapid expansion of aquaculture worldwide, pressure on fisheries exploited for FM and FO production has remarkably increased, reaching and, on occasion, exceeding their ecological sustainability limit. This prompted interest in exploring alternative ingredients for aquaculture and, currently, raw materials derived from land animals or plants are commonly used to partly or totally replace FM and/or FO in aquafeeds [11,12,13]. With respect to FO alternatives, oils derived from microalgae and transgenic oilseed crops have become available in recent years, representing promising sources of the n-3 LC-PUFAs [14,15,16]. Currently, the use of vegetal oils (VOs) as substitutes for FO in fish feed has become a widely extended practice [9,17,18]. However, the use of non-marine ingredients is often associated with a loss of nutritional value of farmed fish products mainly due to decreased contents of LC-PUFAs, especially EPA and DHA, which are absent or at low levels in non-marine ingredients. Biomasses derived from low trophic marine crustaceans, such as krill and copepods, as well as the ingredients derived from their processing, have been demonstrated to be excellent sources of essential nutrients including n-3 LC-PUFAs and thus arise as promising alternative raw materials for aquafeed formulation [19,20,21]. However, the exploitation of wild stocks for the production of crustacean-derived ingredients poses the same ecological sustainability issues alluded to above for reduction fisheries. Alternatively, mass production of marine crustaceans appears to be a reasonable strategy to address such a sustainability hurdle, particularly with species of low trophic level with high nutritional value (i.e., high n-3 LC-PUFAs) and culture performance output. 

Gammarids, crustacean amphipods, are aquatic invertebrates that are abundant in benthic communities in virtually all aquatic environments [22,23]. Previous studies have shown that gammarids’ nutritional profiles are characterised by a high protein content, low levels of carbohydrates, and relatively high contents of n-3 LC-PUFAs [24,25,26,27,28,29]. Such characteristics, along with the possibility to establish high density cultures [25,30,31], have prompted interest for using gammarids in aquaculture [32]. Importantly, some investigations reported on the ability of gammarids to be used in a wide range of sidestreams, in bioindustries such as aquaculture, forestry and agriculture [28,30,31,33]. Consequently, gammarids arise as promising candidates to apply circular economy principles by which sidestreams can be utilised for the production of biomass with high nutritional value. Moreover, in the scenario of a limited availability of marine ingredients alluded to above, it is important to elucidate whether marine gammarids are able to bioconvert fatty acids (FAs) present in sidestreams into the high-value physiologically essential LC-PUFAs.

The ability of animals for endogenous production (biosynthesis) of LC-PUFAs depends upon the gene repertoire and function of two types of enzymes [6,34]. On one hand, front-end desaturases (Fad) introduce double bonds (unsaturations) into polyunsaturated fatty acid (PUFA) substrates; on the other hand, elongation of very long-chain fatty acid (Elovl) proteins (or commonly known as “elongases”) catalyse the usually rate-limiting reaction (condensation) in the FA elongation pathway and results in the extension of the pre-existing FA in two carbons [35]. To the best of our knowledge, no studies have reported on the molecular and functional characterisation of *fad* and *elovl* genes from gammarids, and the LC-PUFA biosynthetic capacity of gammarids has been only inferred from feeding experiments assessing the impact of dietary FA on body FA composition [24,25,26,28,33]. While the presence of Fad-like enzymes in crustaceans remains to be clarified [6], *elovl* genes with roles in LC-PUFA biosynthesis have been recently reported in crabs such as the mud crab *Scylla paramamosain* [36], the orange mud crab *Scylla olivacea* [37,38] and the swimming crab *Portunus trituberculatus* [39]. To date, three different elongases named Elovl4, Elovl6 and Elovl7 have been reported in crustaceans [36,37,38,39]. While the vertebrate Elovl4 has been demonstrated to elongate PUFA substrates, Elovl6 and Elovl7 have not been reported to play a role in LC-PUFA biosynthesis of vertebrates [1,35], suggesting functional diversification of animal Elovl [6]. Our overall aim is to elucidate the LC-PUFA biosynthetic pathways in gammarids in order to identify species with a high capacity to convert short-chain FA compounds available in various sidestreams potentially used as feed into high nutritional value LC-PUFAs. Specifically, the present study aimed to characterise molecularly and functionally the set of *elovl* genes identified in *Echinogammarus marinus*, a gammarid species in which capacity for LC-PUFA endogenous production was previously reported [33]. We here provide compelling evidence showing that *E. marinus* possess three *elovl* genes, named Elovl4, Elovl6 and Elovl1/7-like, with roles in LC-PUFA biosynthesis.

## 2. Results

### 2.1. Phylogeny of the E. marinus Elovl

**Figure 1:**
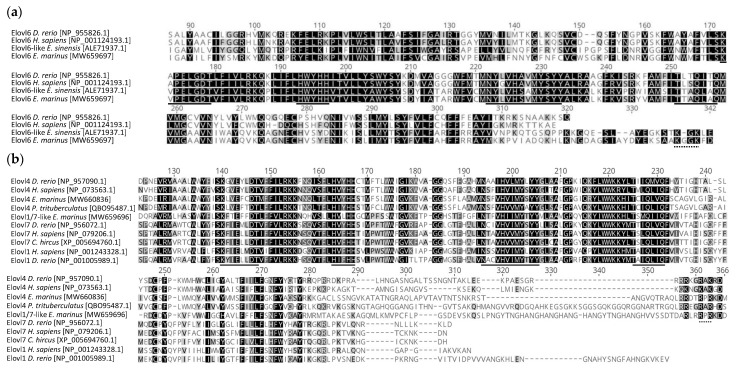
Alignments of the partial deduced amino acid (aa) sequences from the *Echinogammarus marinus* (**a**) SFA/MUFA like elongase *elovl6* (MW659697) and (**b**) PUFA-like elongases *elovl4* (MW660836) and *elovl1/7*-like (MW659696), with several elongases already characterised from vertebrates and crustaceans including the swimming crab *P. trituberculatus*. Conserved domains KXXEXXDT, NXXXHXXMYXYY and TXXQXXQ, and the histidine box (HXXHH) are underlined (solid line). Additionally, aa residues corresponding to putative endoplasmic reticulum retention signal have a dotted underline.

Our search strategy enabled us to identify three *elovl* sequences from *E. marinus*, which were homologous to the three Elovls found in the amphipod reference genome from *Hyalella azteca* [40]. The newly cloned *E. marinus elovl4* cDNA (deposited in GenBank database with accession number MW660836) has an open reading frame (ORF) of 960 base pairs (bp), encoding a putative protein of 319 amino acids (aa). The *E. marinus elovl6* cDNA has an ORF of 1029 bp (MW659697) that translates to a putative protein of 342 aa residues, whereas *elovl1/7*-like contains an ORF of 1077 bp (MW659696) that encodes for a putative protein of 358 aa. The putative aa sequences deduced from the *E. marinus elovl* have some of the distinctive characteristics of fatty acyl elongases, such as the conserved domains KXXEXXDT, NXXXHXXMYXYY and TXXQXXQ, a histidine box (HXXHH) and a specific number of transmembrane-spanning regions (Figure 1). More specifically, prediction of transmembrane-spanning domains showed that the deduced aa sequences of the three *E. marinus* Elovls contained seven transmembrane-spanning regions (Figure S1A–C). The histidine box (HXXHH), a common feature shared with desaturase and hydrolase enzymes, and crucial for the coordination of electron exchange during FA elongation [41,42], was found in all three *E. marinus* Elovls (Figure 1). However, the sequence of the N-terminal side of the histidine box of the three *E. marinus* Elovls presented some noteworthy differences. On one hand, Elovl4 and Elovl1/7-like from *E. marinus* had, respectively, a glutamine (Q) and a histidine (H) at position -5 preceding their histidine boxes; on the other hand, the *E. marinus* Elovl6 had a proline (P) at position -5 and a lysine (L) at position -4 (Figure 1). Subcellular location analysis revealed that these three elongases are located in the endoplasmic reticulum (ER) membrane (Table S1A,B).

**Figure 2:**
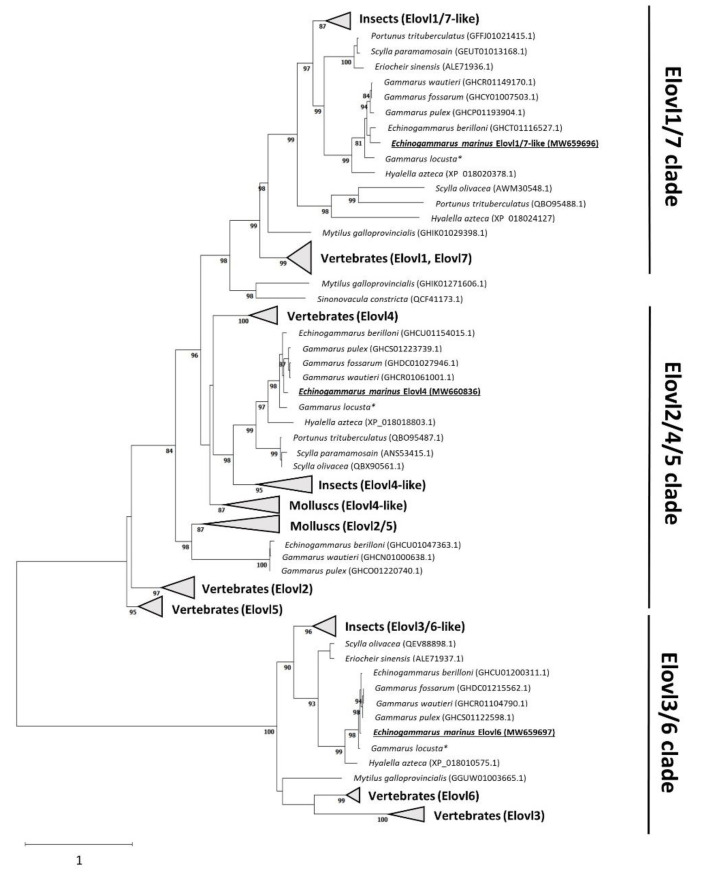
Phylogenetic tree comparing gammarid Elovl with several elongases retrieved from other crustaceans, as well as molluscs and vertebrates. There are three different clades that grouped the majority of the sequences analysed, namely Elovl1/7 clade, Elovl2/4/5 clade and Elovl3/6 clade. Two sequences that do not correspond to any of the previously defined clusters are PUFA elongases found in *Mytilus galloprovincialis* and *Sinonovacula constricta* [43]. The protein evolutionary model used was LG4X [44]. The tree was constructed using the maximum likelihood method by randomized axelerated maximum likelihood (RAxML) within the CIPRES external server. Confidence in the resulting phylogenetic tree branch topology was measured by bootstrapping through 1000 iterations. The transfer distance bootstrap support value (%) is given in each node. Values lower than 80% are not shown. Accession numbers according to the NCBI database are given for each sequence. * Elovls from *Gammarus locusta* were retrieved from blasting the transcriptome assembled by Neuparth et al. [45].

In order to elucidate the orthology of the newly cloned *elovl* from *E. marinus*, a phylogenetic tree was constructed to compare them with Elovl proteins from a variety of animal groups, including both invertebrates and vertebrates (Figure 2). Phylogenetic analysis of the aa sequences showed that the putative *E. marinus* Elovl4 grouped together with Elovl4 from the crustaceans *P. trituberculatus* and *S. olivacea*, as well as vertebrate Elovl4. The *E. marinus* Elovl6 protein grouped closely to Elovl6 sequences from other crustaceans such as the Chinese mitten crab *E. sinensis* and more distantly from their vertebrate orthologues. With respect to the phylogeny of the Elovl1/7-like retrieved from *E. marinus*, the putative protein clustered together with a group of as yet unnamed putative Elovl proteins from other crustaceans like *P. trituberculatus* and *S. paramamosain*, themselves being closely related to *H. azteca* (XP_018024127.1) and *S. olivacea* (AWM30548.1) that were originally annotated as “Elovl7-like”. Three *elovl2/5*-like sequences were identified from freshwater gammarids including *Echinogammarus berilloni*, *Gammarus waitieri* and *Gammarus pulex*, but no homologous sequences of this *elovl* type could be found in transcriptomic databases from either *E. marinus* or *G. locusta*.

All putative Elovl proteins found in *E. marinus* were further confirmed to exist in other gammarids including both freshwater and marine species from the *Gammaridae* family (Figure 2). Given the close taxonomic relationship between *Echinogammarus* and *Gammarus* species, the Elovl proteins from the different gammarid species shared a high homology and therefore clustered together in the tree (Figure 2).

### 2.2. Roles of the E. marinus Elovl4, Elovl6 and Elovl1/7-like in LC-PUFA Biosynthesis 

**Figure 3:**
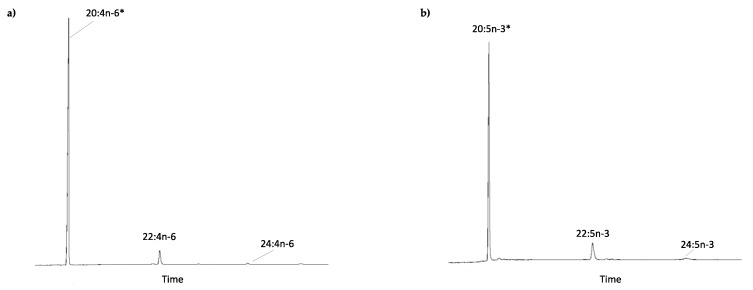
Functional characterisation of the *E. marinus* Elovl1/7-like activity towards C_20_ PUFA substrates. Yeast (*S. cerevisiae*) expressing the *E. marinus elovl1/7*-like gene was grown in the presence of (**a**) arachidonic acid (ARA) (20:4n-6) and (**b**) eicosapentaenoic acid (EPA) (20:5n-3). Substrates (*) and their corresponding elongation products are indicated in each panel.

The role of the *E. marinus* Elovl proteins in LC-PUFA biosynthesis was investigated by expressing their ORFs in yeast. Transgenic yeast expressing each of the *E. marinus elovl* sequences were grown in the presence of exogenously supplied PUFA substrates including linoleic acid (LA, 18:2n-6), α-linolenic acid (ALA, 18:3n-3), γ-linolenic acid (GLA, 18:3n-6), stearidonic acid (SDA, 18:4n-3), ARA (20:4n-6), EPA (20:5n-3), docosatetraenoic acid (DTA, 22:4n-6), docosapentaenoic acid (DPA, 22:5n-3) and DHA (22:6n-3). The elongase conversions are shown in Table 1. For the *E. marinus* Elovl4, the results showed that this elongase has the ability to elongate all PUFA substrates to the corresponding 2-carbon longer products (Table 1). Conversions of C_18_ and C_20_ substrates were all above 1%, whereas conversions towards C_22_ were below 1% (Table 1). A second elongation product was detected when 18:4n-3, 20:5n-3, 20:4n-6 and 22:5n-3 were used as substrates, resulting in the production of 22:4n-3, 24:5n-3, 24:4n-6 and 26:5n-3 (Table 1). The *E. marinus* Elovl6 was able to elongate only C_18_ PUFA, with conversions to the corresponding C_20_ products being below 0.4 % in all cases (Table 1). Functional characterisation of the *E. marinus* Elovl1/7-like revealed that, like Elovl4, this enzyme was able to elongate all substrates assayed (Table 1). It is worth mentioning that conversions towards the C_20_ substrates EPA and ARA were relatively high (13.17 and 5.75 %, respectively), and also a second elongation product was detected in both cases (Figure 3). However, unlike Elovl4, the *E. marinus* Elovl1/7-like did not produce 26:5n-3. 

## 3. Discussion

There exists a growing interest in using gammarids in aquaculture associated with their nutritional profiles, characterised by relatively high contents of n-3 LC-PUFA [24,25]. Moreover, gammarids have shown an ability to be grown on a varied range of sidestreams from bio-based industries [28,30,31,33]. Recently, Alberts-Hubatsh et al. [33] reported that the gammarids *E. marinus* and *G. locusta* had some capacity for “trophic upgrading”, implying that these crustaceans can bioconvert FA present in sidestreams from agriculture into high nutritional value LC-PUFA [33]. However, the actual enzymes accounting for such FA conversions remained to be elucidated and, therefore, this study contributes to investigating the role that endogenous Elovls play in gammarids’ trophic upgrading capacity. We herein report on three elongases with putative functions in the LC-PUFA biosynthesis of gammarids, and demonstrate that the relatively high LC-PUFA contents of gammarids grown on agriculture sidestreams can account for the endogenous enzymatic capacity existing in gammarids themselves. 

Our phylogenetic analysis revealed a well-conserved pattern among gammarids, consisting of three Elovl proteins that have been here referred to as Elovl4, Elovl6 and Elovl1/7-like. The phylogenetic analysis clearly established that both *elovl4* and *elovl6* found in multiple gammarids are closely related to the Elovl4 and Elovl6 present in higher metazoans [1,35], and previously characterised in other non-gammarid crustaceans like the mud crab *S. paramamosain* [36], the swimming crab *P. trituberculatus* [39] and the orange mud crab *S. olivacea* [38]. Interestingly, we named a further *elovl* as “*elovl1/7*-like” since the phylogenetic analyses illustrated that this elongase grouped closely with the vertebrate Elovl1 and Elovl7 subfamilies. Moreover, our results showed that this elongase is closely related to an Elovl named as “Elovl7-like” for *S. olivacea* [37]. It is important to note that some invertebrates such as molluscs possess an elongase named Elovl2/5 [43,46,47,48], which represents an ancestor elongase that gave rise to the Elovl2 and Elovl5 protein families present in vertebrates [49]. Like Elovl2 and Elovl5 [35], the mollusc Elovl2/5 has well-established roles in the elongation of PUFAs [43,46,47,48]. Our *elovl*-like sequence search strategy identified three putative *elovl2/5* sequences from freshwater gammarids, namely *Echinogammarus berilloni*, *Gammarus waitieri* and *Gammarus pulex*. However, multiple attempts to isolate a partial sequence of this elongase from marine gammarids including *E. marinus* and *G. locusta* did not result in any conclusive result, suggesting that these elongases could be absent from genomes of these species or, alternatively, are expressed at relatively low levels compared to those of the elongases characterised in this study. 

We further characterised the sequences of the *elovl* genes present in the marine gammarid *E. marinus*, a species in which capacity for trophic upgrading of dietary FA had been previously reported [33], and is now also confirmed to be a valid representative of marine gammarids as it possesses at least one copy of the *elovl4*, *elovl6* and *elovl1/7*-like genes. The newly cloned *elovl4*, *elovl6* and *elovl1/7*-like from *E*. *marinus* have all the distinctive characteristics shared by Elovls [41] and were further confirmed to exist in orthologues from *S. paramamosain, S. olivacea* and *P. trituberculatus* [36,38,39]. Such Elovl characteristic traits include the conserved domains KXXEXXDT, NXXXHXXMYXYY, TXXQXXQ, a histidine box (HXXHH) and a specific number of transmembrane-spanning regions. However, the above-described differences in the residues located in the N-terminal side of the histidine box between the *E. marinus* Elovl4 and Elovl1/7-like and Elovl6 suggest different putative functions according to the categorisation of PUFA vs. non-PUFA elongases proposed by Hashimoto et al. [42]. Clearly, our results established that both Elovl4 and Elovl1/7-like from *E. marinus* and other gammarids, such as *G. locusta*, have the diagnostic residues in the surrounding region of the histidine box that are a common feature of PUFA elongases [42]. On the contrary, the gammarid Elovl6 contains the diagnostic residues that are typically found in Elovls involved in elongation of saturated and monounsaturated fatty acids [42]. The predicted subcellular localisation results showed that all three Elovls are located in the ER, the cellular site where LC-PUFA biosynthesis takes place [1,35,41]. Retention in the ER is crucial for Elovl to exert its function and, consistently, the *E. marinus* Elovl4 and Elovl1/7-like, but not Elovl6, have the RXR motif that is also associated with ER retention [50] and predicted in the *P. trituberculatus* Elovl4 [39]. Collectively, the molecular characterisation results showed that the *E. marinus* Elovl4 and Elovl1/7-like have distinctive features with respect to Elovl6, which can partly account for the substrate specificities revealed in our functional assays in yeast. 

The functional characterisation of the herein studied *E. marinus* Elovls revealed that these enzymes, particularly Elovl4 and Elovl1/7-like, play roles in the LC-PUFA biosynthesis of gammarids, which likely account for the trophic upgrading capacity of these crustaceans [33]. Indeed, the gammarid Elovl4 and Elovl1/7-like were able to utilise a variety of C_18-22_ PUFAs as substrates and convert them into longer products, including multiple LC-PUFAs. On the contrary, the chain length of PUFA substrates recognised by the *E. marinus* Elovl6 was limited to C_18_ and with relatively low conversions throughout. While our results do not allow us to completely rule out a putative role of the *E. marinus* Elovl6 in PUFA elongation, the restricted elongation capacity towards C_18_ PUFA, along with the abovementioned sequence characteristics of non-PUFA Elovl [42], suggests that Elovl6 does not play a relevant role in LC-PUFA biosynthesis in gammarids. Importantly, the elongation abilities contained within the gammarid Elovl4 and Elovl1/7-like enable these crustaceans to catalyse all elongation reactions involved in the LC-PUFA biosynthetic pathway, even in the absence or low expression of the PUFA elongase *elovl2/5* in marine species such as *E. marinus* and *G. locusta*, as pointed out above. The ability of the gammarid Elovl4 and Elovl1/7-like to produce 24:5n-3 from 22:5n-3, but also from 20:5n-3, suggests that these elongases can contribute to the biosynthesis of DHA via the Sprecher pathway, requiring 24:5n-3 as an intermediate for a Δ6 desaturase to produce 24:6n-3, which is subsequently β-oxidised to DHA (22:6n-3) [51]. This key metabolic pathway is found in some mammals [51], fish [52] and molluscs [43], where Δ6 desaturases that are able to convert 24:5n-3 into 24:6n-3 have been reported. However, to the best of our knowledge, no front-end desaturases from gammarids having such capacity have been yet reported in the literature. While further studies aiming to characterise the gene complement and function of front-end desaturases from gammarids are needed, the current evidence suggest that it is unlikely that DHA can be produced via the Sprecher pathway in gammarids. Likewise, biosynthesis of other physiologically important LC-PUFA such as EPA and ARA appears to not be possible either, unless the existence of front-end desaturases, particularly Δ5 desaturases, is demonstrated to exist in gammarids. Rather than biosynthetic products, however, EPA and ARA appear to be metabolic precursors in gammarids, according to the results of the yeast assays showing that these compounds can be efficiently elongated to the corresponding C_22_ elongation products. Conversion of EPA to 22:5n-3 (DPA) was particularly high (13.17%) for Elovl1/7-like, and considering the low concentration of DPA in detritus that gammarids naturally feed on, it is tempting to speculate that part of the DPA found in wild caught *E. marinus* [33] derives from biosynthesis.

Beyond its role in LC-PUFA biosynthesis, the gammarid Elovl4 can also participate in the biosynthesis of the so-called very long-chain (>C_24_) PUFAs (VLC-PUFAs). Our functional characterisation assays showed that transgenic yeast expressing the *E. marinus elovl4* were able to produce 26:5n-3 when 22:5n-3 was supplied as a substrate. Such elongation capacity was found to be unique among the three gammarid elongases characterised in the present study, since neither the *E. marinus* Elovl6 nor Elovl1/7-like were able to synthesise VLC-PUFAs. These results are consistent with those reported previously for the crab *P. trituberculatus* Elovl4 [39] and illustrate putative roles of crustacean Elovl4 in the biosynthesis of VLC-PUFAs as occurs for vertebrates [1,41,52,53], as well as other invertebrates such as molluscs [43,46,54]. Vertebrate Elovl4 is widely expressed in the central nervous system (CNS) and photoreceptors of the retina [53] and, in fish, pineal gland [55], and hence it is believed to play pivotal roles in brain functioning and photoreception by providing VLC-PUFAs that guarantee normal function. Analysis of VLC-PUFAs from natural samples is technically challenging [56,57] and it can be only accurately performed on lipid samples prepared from specific polar lipid fractions collected from key tissues such as the CNS or retina. For that reason, it was not possible to clarify whether the ability of the gammarid Elovl4 for the biosynthesis of 26:5n-3 shown here occurs in vivo. 

In conclusion, the research described in this work demonstrates that gammarids possess at least three distinct *elovl* genes, namely *elovl4*, *elovl6* and *elovl1/7*-like, with putative roles in the biosynthesis of LC-PUFAs. Molecular and functional characterisation of the set of *elovl* sequences from the marine gammarid *E. marinus* revealed that gammarids’ Elovl4 and Elovl1/7-like are PUFA elongases with affinity towards PUFA substrates ranging from C_18_ to C_22_, and account, by themselves, for all the elongation reactions required for LC-PUFA biosynthesis from C_18_ biosynthetic precursors. On the contrary, the gammarid Elovl6 sequence contained characteristics typically found in non-PUFA elongases which, along with its elongase capacity being restricted to C_18_ PUFA substrates, indicated that this enzyme does not play major roles in LC-PUFA biosynthesis in gammarids. Elovl4 was the sole Elovl found in gammarids with the ability to produce PUFAs of up to 26 carbons. Overall, the present study provides insight into the endogenous machinery enabling gammarids to bioconvert dietary fatty acids into high nutritional value LC-PUFAs. 

## 4. Materials and Methods

### 4.1. Molecular Cloning of Full-Length cDNAs of Three Elovls from E. marinus

Several *E. marinus* individuals collected from the wild in the intertidal zone in Trondheim, Norway, were preserved in RNAlater (Thermo Fisher Scientific, Waltham, MA, USA) and sent to the facilities of the Instituto de Acuicultura Torre de la Sal, Spain, for further analysis. Total RNA was extracted from one single whole individual using the Maxwell^®^ instrument and the Maxwell^®^ 16 LEV simplyRNA Tissue Kit (Promega, Madison, WI, USA) following the manufacturer’s instructions. First strand complementary DNA (cDNA) was synthesised from 2 µg of total RNA using Moloney Murine Leukemia Virus Reverse Transcriptase (M-MLV RT) (Promega) following the manufacturer’s instructions.

The strategy to retrieve the *elovl* sequences from *E. marinus* was established by considering that this species possesses the same *elovl* gene complement as *Hyalella azteca*, a closely related amphipod species with a reliable genome annotation (www.hgsc.bcm.edu/arthropods/hyalella-azteca-genome-project (accessed on 10 September 2019); www.ncbi.nlm.nih.gov/bioproject/243935 (accessed on 10 September 2019)). Searches for *elovl* homologues within the *H. azteca* genome resulted in the identification of four sequences: 1) gb|XM_018163314.1|, *H. azteca* elongation of very long-chain fatty acids protein 4-like; 2) gb|XM_018155086.1|, *H. azteca* elongation of very long-chain fatty acids protein 6-like; 3) gb|XM_018168638.1|, *H. azteca* elongation of very long-chain fatty acids protein 7-like; and 4) gb|XM_018164888.1|, *H. azteca* elongation of very long-chain fatty acids protein AAEL008004-like. Preliminary analysis of the sequences suggested that sequence 3) did not translate to any potentially functional putative elongases in *E. marinus*, nor in any other gammarid. Therefore, only sequences 1) “*elovl4”*; 2) “*elovl6”*; and 4), which we hereafter refer to as “*elovl1/7*-like”, were used for further analyses. The *H. azteca elovl*-like sequences were used as queries to run BLAST searches for homologous sequences within the Transcriptomic Shotgun Assembly (TSA) database of the *Gammarus* BioProject (www.ncbi.nlm.nih.gov/bioproject/PRJNA497972 (accessed on 10 September 2019)) as follows. First, *H. azteca elovl4* (gb|XM_018163314.1|) was used to search homologous sequences from gammarid species available at NCBI (www.ncbi.nlm.nih.gov (accessed on 10 September 2019)), which included several gammarid species such as *Echinogammarus berilloni* (GHCU01154015.1), *Gammarus fossarum* (GHDC01027946.1) and *Gammarus pulex* (GHCS01223739.1). Alignment of these sequences (Geneious Prime Software, Version 2019.0.3, www.geneious.com (accessed on 10 September 2019)) [58] enabled the design of the degenerate primers EM_ELO4_F1 and EM_ELO4_R2 on conserved regions (Table 2). A polymerase chain reaction (PCR) using a high-fidelity polymerase (Phusion Green High-Fidelity DNA Polymerase, Thermo Fisher Scientific) was performed in order to amplify the first DNA fragment of the *E. marinus* putative *elovl4.* Primer sequences and PCR conditions are given in Table 1. A PCR product of the expected size (~350 bp) was obtained and then purified on a 1% (*w*/*v*) agarose gel using the Wizard^®^ SV Gel and PCR Clean-Up System (Promega). The identity of the peak was confirmed by DNA sequencing (DNA Sequencing Service, IBMCP-UPV, Valencia, Spain). Subsequently, gene-specific primers based on the first fragment sequence of the *E. marinus* putative *elovl4* were designed in order to obtain the full-length open reading frame (ORF) sequence by rapid amplification of cDNA ends (RACE) PCR (FirstChoice^®^ RLM-RACE Kit, Thermo Fisher Scientific). A two-round (nested) PCR using the *Taq* Polymerase GoTaq^®^G2 Green Master Mix (Promega) was performed. Briefly, the first round of the 3’ RACE PCR was run with the gene-specific primer EM_ELOVL4_F3 (Table 2) and the primer 3’ RACE Outer (provided by the kit), and *E. marinus* 3’RACE cDNA as a template. The second-round PCR was performed using the gene-specific primer EM_ELOVL4_F5 (Table 2) and the primer 3’RACE Inner (provided by the kit), and the first-round PCR product as a template. A 3’ RACE PCR product was obtained, which was subsequently purified and sequenced as above. 

In order to obtain the 5’ end of the *E. marinus elovl4* ORF, a BLASTn (Megablast) was performed using the partial *elovl4* cDNA generated previously as a query against the TSA database of the *Gammarus* BioProject. We identified a high identity sequence from *Marinogammarus marinus* (GHCW01145927.1), a former species name for *E. marinus* [59]. This sequence (GHCW01145927.1), containing part of the 5’ untranslated region (5’UTR) and the 5’ end of the ORF, aligned with the 3’ end of the partial first fragment *elovl4* sequence obtained in previous steps. Gene-specific primers designed in the 5’UTR (EM_ELOVL4_ORF_U5F) and the 3’UTR (EM_ELOVL4_ORF_U3R) were used to obtain a 1246 bp product by running a PCR (Phusion Green High-Fidelity DNA Polymerase), which encompassed the ORF of the putative *E. marinus elovl4*. The PCR product was purified and sequenced as described above. The ORF sequence was predicted using the NCBI ORF Finder tool (www.ncbi.nlm.nih.gov/orffinder (accessed on 17 September 2019)).

For the cloning of the *E. marinus elovl6* and *elovl1/7*-like ORF, we identified contig assemblies containing full-length sequences of the ORF by searching within the *M. marinus* (or *E. marinus*) BioSample (SAMN10259948). Briefly, the *H. azteca* XM_018155086.1 (*elovl6*) and XM_018164888.1 (*elovl1/7*-like) sequences were translated to their corresponding protein sequences and used as queries for TBLASTn searches against the *E. marinus* BioSample (SAMN10259948). These searches allowed the identification of the highly homologous sequences GHCW01079894.1 and GHCW01079198.1, containing, respectively, the putative *elovl6* and *elovl1/7*-like from *E. marinus*. Due to the large size of these sequences (~5.7 to 6.5 Kb), a preliminary analysis was performed in order to predict the ORF sequences for the putative elongases (NCBI ORF Finder). The predicted proteins were subsequently analysed using the Pfam online tool (https://pfam.xfam.org/ (accessed on 15 October 2019)) and those containing the complete “ELO” domain (pfam 01151) were selected as putative Elovls and considered for further analysis. Primer pairs for each target gene (*elovl6* or *elovl1/7*-like) were designed in the putative 5’ and 3’UTR (Table 2), and subsequently used for PCR amplification as described previously to obtain PCR products of 1405 bp for *elovl6*, and 1233 bp for the *elovl1/7*-like.

### 4.2. Sequence and Phylogenetic Analysis of the E. marinus Elongases

The putative aa sequences of the three newly cloned *elovl* from *E. marinus* were predicted using the ORF Finder tool available from NCBI. The transmembrane regions and protein domains of the *E. marinus* Elovl were predicted using the TMHMM online tool (www.cbs.dtu.dk/services/TMHMM/ (accessed on 18 October 2019)). Moreover, the subcellular location of the newly cloned elongases was predicted using the DeepLoc 1.0 online tool (www.cbs.dtu.dk/services/DeepLoc/ (accessed on 29 March 2021)). A phylogenetic analysis comparing the Elovl aa sequences from model vertebrates such as *Danio rerio* and *Mus musculus*, as well as Elovl protein sequences from crustacean species such as Elovl4 from the swimming crab *P. trituberculatus* (QBO95487.1), and Elovl4 (QBX90561.1), Elovl6 (QEV88898.1) and Elovl7 (AWM30548.1) from the orange mud crab *S. olivacea*, was performed. In addition, several putative Elovl protein sequences from other crustacean species, including gammarids from the genus *Gammarus* and other amphipods, were retrieved and also included in the phylogenetic analysis. Briefly, 1) orthologues for the *E. marinus* Elovl sequences were retrieved by tblastn (NCBI) within the *Gammarus* TSA database (PRJNA497972), 2) putative Elovl sequences were analysed using the Pfam online tool and the ones containing the “ELO” domain (pfam 01151) were selected as candidates to be included in the phylogenetic analysis. The phylogenetic tree and all the corresponding analyses were performed using the CIPRES platform [60] (https://www.phylo.org/ (accessed on 29 March 2021)). Briefly, first, a MAFFT alignment and subsequent trimming (TrimAI) were performed. Then, a model test was run in order to select the best evolutionary model for aa substitution. Finally, randomized axelerated maximum likelihood (RAxML) was performed in order to build the phylogenetic tree and the LG4X was used as an evolutionary model [44]. Confidence in the resulting phylogenetic tree branch topology was measured by bootstrapping through 1000 iterations, following the transfer distance bootstrap approach [61,62].

### 4.3. Functional Characterisation of the E. marinus Elovl by Heterologous Expression in Yeast

The ORF sequences of the three *E. marinus elovl* sequences were cloned into the yeast expression vector pYES2 (Invitrogen, Carlsbad, CA, USA). Briefly, primers containing *Hind* III (forward) and *Xba* I (reverse) restriction sites were designed for amplification of the whole ORF of the three *E. marinus elovl* genes (Table 2). The PCRs were performed using the high-fidelity polymerase Phusion Green High-Fidelity DNA Polymerase (Thermo Fisher Scientific), with conditions used for each gene detailed in Table 1. Then, PCR products were purified as above and subsequently digested with the corresponding restriction enzymes before being ligated into similarly restricted pYES2. Ligation reactions were transformed into the *E. coli* competent TOP10^TM^ strain (Invitrogen) and positive colonies were grown overnight in Luria–Bertani (LB) broth containing ampicillin (100 µg/mL). Next, plasmids were purified using a GenElute™ Plasmid Miniprep Kit (Sigma-Aldrich, St Louis, MO, USA) following the manufacturer’s instructions prior to DNA sequencing (IBMCP-UPV) in order to confirm the sequence correctness of the inserts.

The plasmid constructs pYES2-Elovl4, pYES2-Elovl6 and pYES2-Elovl (Elovl1/7-like) were transformed into *Saccharomyces cerevisiae* competent cells (strain INVSc1) (Invitrogen). The recombinant yeast were selected on *S. cerevisiae* minimal medium minus uracil (SCMM^−ura^) agar plates for 3 d at 30 °C. The culture of recombinant yeast was carried out as described in detail by Jin et al. [63]. Briefly, one single recombinant colony from each transformation (*E. marinus elovl* pYES2 constructs) was grown in SCMM^-ura^ broth for 2 d at 30 °C to produce a bulk culture with an OD600 of 8–10. Subsequently, an appropriate volume of the yeast bulk cultures was inoculated in 5 mL of SCMM^-ura^ broth contained in individual 150 mL Erlenmeyer flasks to provide an OD600 of 0.4. The recombinant yeast in each Erlenmeyer flasks was grown for 4 h at 30 °C and under constant shaking (250 rpm) until they reached an OD600 of ~1, at which point the transgene expression was induced by supplementing the culture media with galactose at 2% (*w*/*v*). Moreover, in order to test the ability of the *E. marinus* Elovl to elongate PUFA substrates, recombinant yeast expressing the *E. marinus elovl4*, *elovl6* and *elovl1/7*-like were supplemented with one of the following PUFA substrates in the growth media: α-linolenic acid (18:3n-3), linoleic acid (18:2n-6), stearidonic acid (18:4n-3), γ-linolenic acid (18:3n-6), eicosapentaenoic acid (20:5n-3), arachidonic acid (20:4n-6), docosapentaenoic acid (22:5n-3), docosatetraenoic acid (22:4n-6) and docosahexaenoic acid (22:6n-3). Final concentrations of the exogenously supplied PUFA substrates were 0.5 mM (C_18_), 0.75 mM (C_20_), 1.0 mM (C_22_) as uptake efficiency decreases with increasing chain length [64]. A control consisting of yeast transformed with the empty pYES2 was run exactly as described above for the three *E. marinus* elongases. Except stearidonic acid (18:4n-3), all FA substrates (>98–99% pure) used for the functional characterisation assays were obtained from Nu-Chek Prep, Inc. (Elysian, MN, USA). Yeast culture reagents including galactose, nitrogen base, raffinose, tergitol NP-40, stearidonic acid (>99%) and uracil dropout medium were obtained from Sigma-Aldrich (St. Louis, MO, USA). The yeast cultures were maintained at 30 °C and under constant shaking (250 rpm) for 2 d until yeast was harvested by centrifugation at 1500 g for 2 min. Yeast pellets were washed twice with 5 mL of ddH_2_O, homogenised in 6 mL of 2:1 (*v*/*v*) chloroform:methanol containing 0.01% (*w*/*v*) butylated hydroxytoluene (BHT, Sigma-Aldrich) as antioxidant and stored at −20 °C for a minimum of 24 h in an oxygen-free atmosphere until further analysis.

### 4.4. Fatty Acid Analysis

The FA composition of yeast containing either the ORF of the *E. marinus* elongases (i.e., transformed with either pYES2-Elovl4, pYES2-Elovl6 or pYES2-Elovl) or empty pYES2 (control) was determined using the method described by Monroig et al. (2013) [65]. Briefly, total lipids were extracted from the homogenised yeast samples using the Folch method [66]. Subsequently, total lipids were used to prepare fatty acid methyl esters (FAME), which were analysed using gas chromatography coupled with mass spectrometry (GC–MS) [55]. The elongation of exogenously supplemented PUFA substrates by the *E. marinus* Elovl4, Elovl6 and Elovl1/7-like was calculated by the proportion of substrate FA converted to elongated FA product(s) as [areas of all products with longer chain than substrate/(areas of all products with longer chain than substrate + substrate area)] × 100.

## Figures and Tables

**Table 1:** Functional characterisation of the *Echinogammarus marinus* Elovl4, Elovl6 and Elovl1/7-like. Conversions of exogenously supplied fatty acid (FA) substrates were calculated according to the formula [areas of all products with longer chain than substrate/(areas of all products with longer chain than substrate + substrate area)] × 100.

FA Substrate	FA Product	Elovl4	Elovl6	Elovl1/7-Like	Activity
18:3n-3	20:3n-3	2.67	0.22	0.29	C18→C20
18:2n-6	20:2n-6	1.32	0.11	0.19	C18→C20
18:4n-3	20:4n-3	1.82	0.33	1.46	C18→C20
	22:4n-3	0.02	nd	0.02	C20→C22
18:3n-6	20:3n-6	1.21	0.35	0.87	C18→C20
20:5n-3	22:5n-3	2.52	nd	13.17	C20→C22
	24:5n-3	0.05	nd	0.17	C22→C24
20:4n-6	22:4n-6	1.61	nd	5.75	C20→C22
	24:4n-6	0.02	nd	0.09	C22→C24
22:5n-3	24:5n-3	0.92	nd	2.16	C22→C24
	26:5n-3	0.09	nd	nd	C24→C26
22:4n-6	24:4n-6	0.46	nd	0.72	C22→C24
22:6n-3	24:6n-3	0.38	nd	0.54	C22→C24

nd, not detected.

**Table 2:** Primer sets and corresponding PCR conditions used in the cloning of the *E. marinus elovl4*, *elovl6* and *elovl1/7*-like genes.

Gene	Aim	Primer Name	Primer Sequence	Cycles	Tm	Extension
**ELOVL4**	1st fragment generation	EM_ELOVL4_ F1	TCTACAACCTTGCTGTCATG	35	55 °C	72 °C (1 min)
		EM_ELOVL4_ R2	TGCACAAAGCTGTTCATCAT			
	3’RACE PCR	3’Outer_Primer	GCGAGCACAGAATTAATACGACTCACTATAGGT12	35	55 °C	72 °C (75 s)
		3’Inner_Primer	CGCGGATCCGAATTAATACGACTCACTATAGGT12			
		EM_ELOVL4_F3	CACGTGTATCACCACTCGAC			
		EM_ELOVL4_F4	GGATTGGAGTCAAGTTTGTGG			
		EM_ELOVL4_F5	CCTGGCGGCAATGATGAACA			
	Full ORF	EM_ELOVL4_ORF_U5F	ATGAATTTAGTGAACAACAA	35	62 °C (10 cycles) 58 °C (25 cycles)	72 °C (1 min)
		EM_ELOVL4_ORF_U3R	CAAGATGCCTGAACTCCCGGT			
	Functional characterisation	EM_FW_ELOVL4_HindIII EM_RV_ELOVL4_XbaI	CCCAAGCTTACAATGGCTGCCTCTGTT CCGTCTAGACTACATGTCCTTTCGAGG	35	62 °C (10 cycles) 58 °C (25 cycles)	72 °C (45 s)
**ELOVL6**	1st fragment generation	EM_ELOVL6_ F1 EM_ELOVL6_ R2	GGCTTCTGGAACTGGATGTT TTCATGTAGGCACGACGGAA	35	55 °C	72 °C (1 min)
	Full ORF	EM_ELOVL6_ORF_U5F EM_ELOVL6_ORF_U3R	CTTTACCACGTTTTACTGGG ACTGGTAGTTTTGTATGCAT	35	62 °C (10 cycles) 58 °C (25 cycles)	72 °C (1 min)
	Functional characterisation	EM_FW_ELOVL_HindIII EM_RV_ELOVL_XbaI	CCCAAGCTTACGATGGCCCTCTCGGAC CCGTCTAGATTAATCGAACTTCCCTCCCT	35	62 °C (10 cycles) 58 °C (25 cycles)	72 °C (45 s)
**ELOVL1/7-LIKE**	1st fragment generation	EM_ELOVL_ F1	GTCATCCACCACGGATGCATG	35	55 °C	72 °C (1 min)
		EM_ELOVL_ R1	GCCTTCACGTAGAAGTTGGAG			
	Full ORF	EM_ELOVL_ORF_U5F	AAAAACGTGTTCTCGGCCAG	35	62 °C (10 cycles) 58 °C (25 cycles)	72 °C (1 min)
		EM_ELOVL_ORF_U3R	GAGGCTTAACTAAAACGAAC			
	Functional characterisation	EM_FW_ELOVL_HindIII EM_RV_ELOVL_XbaI	CCCAAGCTTAAGATGGCGGGTACAGCA CCGTCTAGATCAGTCGTCCTTCCGAGG	35	62 °C (10 cycles) 58 °C (25 cycles)	72 °C (45 s)

## Data Availability

The data presented in this study are included in the corresponding sections throughout the manuscript. Sequences of the newly characterised *Echinogammarus marinus elovl* genes were deposited in GenBank (www.ncbi.nlm.nih.gov/genbank/) (accessed on 10 September 2019).

## Supplementary Materials

The following are available online at https://www.mdpi.com/article/10.3390/md19040226/s1, Figure S1: Predicted transmembrane-spanning regions of *E. marinus* elongases, Table S1: Subcellular localisation prediction of the three Elovl proteins from *E. marinus* and Elovl type determination.
