# Supplementary material for: Biosynthesis of Long-Chain Polyunsaturated Fatty Acids in Marine Gammarids: Molecular Cloning and Functional Characterisation of Three Fatty Acyl Elongases
Source: Mar Drugs. 2021 Apr 16;19(4):226. doi: 10.3390/md19040226 (PMC8073319; doi:10.3390/md19040226)
Supplement: Supplementary file 1 [file marinedrugs-19-00226-s001.zip › marinedrugs-1152074-supplementary.pdf]

a)

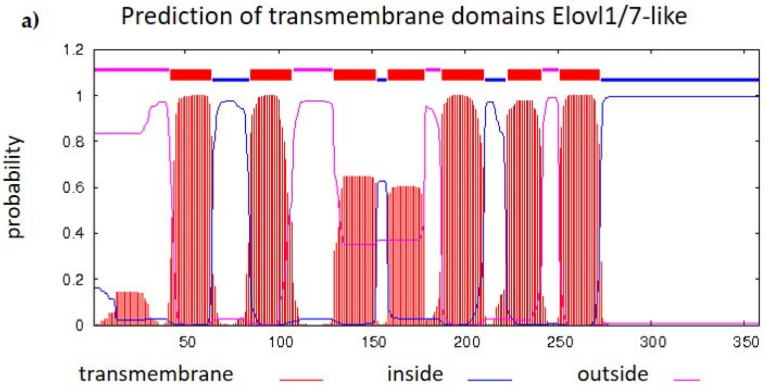

TMD 42 64
TMD 85 107
TMD 130 152
TMD 159 178
TMD 188 210
TMD 223 241
TMD 251 272

b)

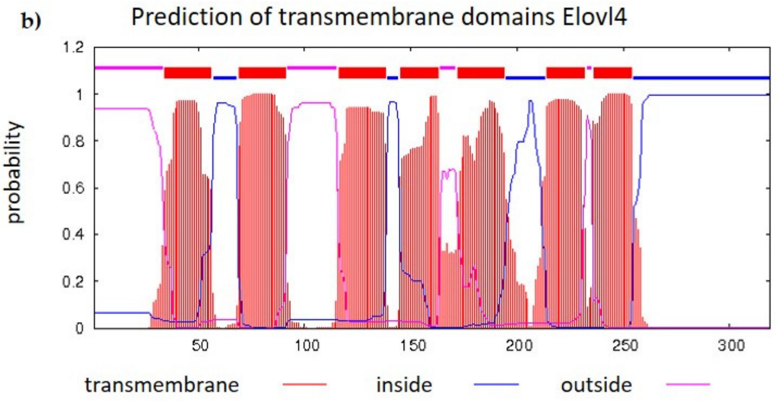

TMD 34 56
TMD 69 91
TMD 116 138
TMD 145 163
TMD 172 194
TMD 214 232
TMD 236 254

c)

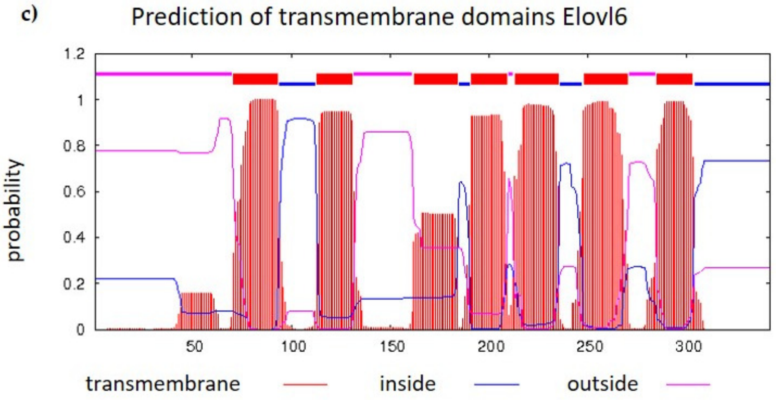

TMD 71 93
TMD 113 131
TMD 162 184
TMD 191 209
TMD 213 235
TMD 248 270
TMD 285 303

Supplementary Figure S1. Predicted transmembrane-spanning domains (TMD) of *E. marinus* **(a)** Elovl1/7-like, **(b)** Elovl4 and **(c)** Elovl6. Inside-Cytosol; Outside-Lumen.

Supplementary Table S1. (a) Subcellular localisation prediction of the three Elovl proteins from E. marinus and (b) Elovl type determination. Prediction was obtained using the DeepLoc 1.0 online tool. Data are represented as % of likelihood.

(a)

	Subcellular localisation (% likelihood)									
	**Endoplasmic reticulum**	**Cell membrane**	**Golgi apparatus**	**Lysosome/ Vacuole**	**Mitochondrion**	**Peroxisome**	**Cytoplasm**	**Nucleus**	**Plastid**	**Extracellular**
**ELOVL1/ 7-LIKE**	79.85	10.74	5.96	3.42	0.02	0	0	0	0	0
**ELOVL4**	87.77	5.75	4.76	1.68	0.02	0	0	0	0	0
**ELOVL6**	81.62	6.71	6.12	5.22	0.17	0.14	0.01	0	0	0

(b)

	Protein type (% likelihood)	
	**Soluble**	**Membrane**
**ELOVL1/7-LIKE**	0	100
**ELOVL4**	0.01	99.99
**ELOVL6**	0.01	99.99
